# Comparison of intravaginal and interstitial brachytherapy in cervical cancer after inadvertent hysterectomy: a retrospective study

**DOI:** 10.1038/s41598-025-99935-4

**Published:** 2025-09-30

**Authors:** Sunil Choudhary, Ankita Pandey, Ankita Singh, Ankur Mourya, Neha Gupta, Syed Mohamed Shajid, Gogul Priean Venkatachalam, Sangita Rai, Sakshi Agarwal, Pitchaikannu Venkatraman, Lalit Mohan Aggarwal

**Affiliations:** 1 Department of Radiotherapy and Radiation Medicine, Institute of Medical Sciences, Banaras Hindu University, Varanasi, 221005 Uttar Pradesh India; 2 Department of Radiation Oncology, Postgraduate Institute of Medical Education & Research, Sector-12, Chandigarh, 160012 India; 3 Department of Radiotherapy, State Cancer Institute, Cancer Hospital, Netaji Subhash Chandra Bose Medical College, Jabalpur, 482003 Madhya Pradesh India; 4 Apex Hospital, Varanasi, 221005 Uttar Pradesh India; 5 Department of Radiation Oncology, Garud Cancer Hospital, Savedi Road, Savedi, Ahmednagar, 414003 Maharashtra India; 6 Department of Obstetrics & Gynaecology, Institute of Medical Sciences, Banaras Hindu University, Varanasi, 221005 Uttar Pradesh India

**Keywords:** Cervical cancer, Hysterectomy, Brachytherapy, Recurrence, Radiotherapy, MUPIT, Biophysics, Gastroenterology, Health care, Oncology

## Abstract

The purpose of this study was to compare intravaginal brachytherapy (IVBT) with interstitial brachytherapy (ISBT) in patients presenting with residual or recurrent disease after inadvertent hysterectomy for cervical cancer. Records of consecutive patients with cervical cancer, registered in the Radiotherapy Outpatient Department (RTOPD) between March 2018 and March 2021, who had inadvertent hysterectomy followed by external beam radiotherapy (EBRT) and IVBT (*n* = 15) or ISBT (*n* = 15) were analyzed retrospectively. All the patients received a dose of 45–50 Gy/25 fractions in 5 weeks by EBRT with concurrent cisplatin. A dose of 6 Gy/fraction/week for two consecutive weeks was prescribed at 5 mm from the surface of the vaginal cylinder in the IVBT arm. Patients in the ISBT arm were treated with 6 Gy/fraction for four fractions over two days with Martinez Universal Perineal Interstitial Template (MUPIT). Overall survival (OS) and disease-free survival (DFS) were the primary endpoints. Acute and late toxicities were the secondary endpoints. Median follow-up (FU) was 24.3 months and 32.8 months in the IVBT and ISBT arms respectively. Most of the patients failed within 12–18 months after brachytherapy. The 3-year OS for IVBT and ISBT arms were 54% and 93% respectively (*p* = 0.011). The 3-year DFS for IVBT and ISBT arms were 42% and 93% respectively(*p* = 0.023). Both the arms had similar acute & late toxicities. ISBT had significantly better survival outcomes in terms of OS, and DFS with similar toxicity as compared to IVBT in patients with residual or recurrent disease after inadvertent hysterectomy for cervical cancer.

**Supplementary Information:**

The online version contains supplementary material available at 10.1038/s41598-025-99935-4.

## Introduction

Cervical cancer remains a significant global health concern, particularly in regions with limited access to screening and treatment modalities^1^. A conservative surgery, conization with pelvic lymph node dissection (PLND) or simple hysterectomy with PLND for low-risk FIGO (2009) IA2-IB1 (maximum size 2 cm) cervical cancers has been well established by ConCerv and SHAPE trials^2,3^. Radical hysterectomy with PLND is the surgical option for cervical cancer FIGO (2019) IB2-IIA1 and lesions more than 2 cm^2^. Anything less than optimal surgery qualifies as an inadvertent hysterectomy^3,4^.

Due to various reasons, inadvertent hysterectomy in cervical cancer is a common practice in most underdeveloped and developing countries^3,5,6^. About 60% of these patients, if left untreated, are destined to recur at the vaginal vault^7^. Adjuvant external beam radiotherapy (EBRT) followed by intravaginal brachytherapy (IVBT), commonly practiced in such cases, results in 5-year overall survival (OS) of 27.9–45%^8–12^.

Brachytherapy offers several advantages, including the delivery of high doses of radiation to the target area while minimizing exposure to surrounding healthy tissues, thus enhancing therapeutic efficacy and reducing treatment-related toxicities^13^. Intravaginal brachytherapy (IVBT), delivered through applicators inserted into the vaginal cavity, has been the standard approach for many years. However, in cases where a hysterectomy has been inadvertently performed, interstitial brachytherapy (ISBT) may be considered as an alternative technique. ISBT involves the insertion of needles directly into the tumor bed, allowing for precise placement of radiation sources and potentially improving outcomes in these challenging scenarios. While both IVBT and ISBT have demonstrated efficacy in the treatment of cervical cancer, comparative studies evaluating their respective roles in the setting of inadvertent hysterectomy are non-existent in the literature. However, existing evidence primarily consists of small retrospective series and single-center experiences, often with heterogeneous patient populations and treatment protocols^7–10^. Therefore, there is a need for a large-scale study to further elucidate the comparative effectiveness and safety profiles of these two brachytherapy techniques in this specific clinical scenario.

This retrospective study was aimed to compare the oncologic outcomes, treatment-related toxicities, and survival rates of patients with cervical cancer who underwent either intravaginal or interstitial brachytherapy following inadvertent hysterectomy. By analyzing data from a single tertiary care center, we sought to provide valuable insights into the optimal management of this challenging patient population and improve clinical decision-making. Hence, we conducted this study to compare the above two brachytherapy techniques concerning survival outcomes and treatment-related toxicities in patients undergoing inadvertent hysterectomy for cervical cancer.

## Materials and methods

### Patient selection

Records of histologically proven cervical cancer patients registered in the Radiotherapy Out-patient Department (RTOPD) from March 2018 till March 2021 were screened for this study and those who met *all* of the following inclusion criteria were taken up for this study:

1. Upfront inadvertent hysterectomy.
2. Residual or recurrent disease at the vault with or without pelvic nodes.
3. No history of any adjuvant treatment before registering with us.
4. Treated with adjuvant EBRT and IVBT or ISBT.

Patients found to have disease outside the pelvis or any metastatic spread were excluded from this study.

### External beam radiotherapy

Patients were simulated in the supine position on a conventional simulator or CT simulator who were to be treated with Tele-cobalt unit and 6 MV Linear Accelerator (LA) respectively. Patients on Tele-cobalt were treated with conventional two parallel opposed fields or 4-field box techniques based on anterior-posterior separation using standard portals. Patients on LA were treated with three-dimensional conformal radiotherapy (3DCRT).

All the patients were treated to a dose of 45–50 Gy/ 25 fractions over 5 weeks. Routine blood tests and clinical examinations were done once a week during EBRT.

### Chemotherapy

Cisplatin was given at 40 mg/m^2^ once a week with EBRT. Patients above 65 years of age were treated with RT alone. Patients with positive pelvic lymphadenopathy were given three cycles of Paclitaxel (175mg/m^2^) and Carboplatin (AUC-5) at 3-week intervals before EBRT.

### Brachytherapy

BT was done one week after the completion of EBRT. We practiced IVBT in these patients till December 2019 and due to the poor treatment outcome with this technique, we switched to ISBT for such patients since January 2020.

#### Intravaginal brachytherapy

A vaginal cylinder of the largest diameter that snugly fitted into the vagina was used for IVBT. Based on our earlier experience with CT and radiograph-based planning for IVBT, we developed pre-defined plans with fixed dwell positions and weightage for vaginal cylinders of various diameters and different treated lengths of the vagina. Therefore, simulation was not performed in these patients after the insertion of the vaginal cylinder applicator. This helped us in reducing the time and treatment costs for our patients.

A proximal 3 cm of the vagina below the lowest extent of the residual disease at the time of BT was treated. A dose of 6 Gy x 2 fractions (one fraction/week) by High dose rate brachytherapy (HDR) was prescribed at 5 mm from the surface of the vaginal cylinder.

#### Interstitial brachytherapy

Martinez Universal Perineal Interstitial Template (MUPIT) with hollow stainless-steel (trocar cut) needles and stylets were used for ISBT. The number of needles required for a particular patient was based on the dimensions of the residual disease evaluated as per the MRI pelvis done before BT. The implant needles were inserted 2 cm beyond the vaginal surface of the vault or the proximal extent of the residual disease to account for the thickness of the vaginal apex and an offset of 1 cm of these needles.

ISBT was done under general or spinal anesthesia. A plain CT scan with a 1 mm slice thickness from the level of the umbilicus till mid-thigh was done in these patients on the day of BT with the applicator in situ. The images were transferred to the Treatment Planning System (OncentraBrachy, v.4.6.0; Elekta AB, Stockholm, Sweden) for delineation of the target, OARs, and BT planning.

Gross tumor volume (GTV) was defined on CT images as a residual disease at the time of BT (GTV_b_). High-risk clinical target volume (HR-CTV) included GTV_b_ with a 5 mm radial margin. A proximal 3 cm of the vagina below the lowest extent of the residual disease was also included in HR-CTV.

HR-CTV included the vaginal vault with a 5 mm radial margin and an upper 3 cm of the vagina for those who had a complete response after EBRT. The rectum, bladder, and sigmoid colon were contoured in each axial slice as OARs^14,15^.

The Inverse Planning Simulated Annealing (IPSA) algorithm was used for dose optimization. The dose was prescribed to HR-CTV with the objective that the dose received by 90% of the volume should be more than 100% of the prescribed dose (D_90_ > 100%). The patients were treated with a dose of 6 Gy/fraction twice daily on the day of application and on the next day for a total of four fractions. The minimum gap between the two fractions was kept as six hours. All the patients were treated with HDR-BT on micro-Selectron version 3.0 (Elekta AB, Stockholm, Sweden).

##### Clinical evaluation and follow-up

Patients were clinically examined once a month for the first six months and three months thereafter. The radiological investigations were done on clinical suspicion of disease recurrence.

### Statistical analysis

The statistical analysis was performed using the software package SPSS version 23 (Chicago, II, USA). The chi-square method was employed to determine the statistical significance between the two treatment arms. Overall survival (OS) and Disease-free survival (DFS) were the primary endpoints. OS was calculated from the date of registration to the date of death due to any cause or date of last follow-up (LFU). DFS was calculated from the date of registration to the date of recurrence or the date of LFU. Acute and late toxicities were the secondary endpoints. The Kaplan-Meier method was used for survival analysis and the log-rank test was used to compare the survival between the two groups. A p-value of less than 0.05 was considered statistically significant.

### Ethics statement

This study wascarried out in accordance with relevant guidelines and regulations. The experimental protocols were reviewed and approved by the Banaras Hindu University human ethical clearance committee. Informed consent was obtained from all subjects and their legal guardians before participation in the study.

## Results

Since we started practicing ISBT in January 2020, we could treat only fifteen patients with the above technique till March 2021. To match the number, we chose the first 15 patients who were treated with IVBT from March 2018 to December 2019. The data analysis was done on 01.04.2024. Patients and tumor characteristics are shown in Table 1. The median age in the ISBT and IVBT groups was 50 years and 52 years, respectively. On the first visit to us, all the patients had gross residual or recurrent disease at the vault and 9 patients had significant pelvic nodes. Adenocarcinoma was found in three patients while the rest had squamous cell carcinoma (SCC). Almost half of the patients in both arms had well-differentiated tumors and one patient in each arm had a poorly differentiated tumor. The histopathological report was incomplete in 80% of patients. Three cycles of Paclitaxel and Carboplatin combination were given before EBRT in nine patients. EBRT was delivered by 2D conventional planning in 12 patients while the rest were treated with 3DCRT. The median duration and dose of EBRT were 38 days and 45 Gy, respectively. The cumulative EQD2 of CTV D90 was 76.25 Gy for ISBT and 60.25 Gy for IVBT arm. The median total RT duration was 58 days and 56 days in the IVBT and ISBT arms, respectively. The majority of patients in both arms received concurrent CRT (Table 1).

**Table 1:** Patient, tumor & treatment characteristics of ISBT and IVBT.

Parameters	ISBT (*n* = 15)	IVBT (*n* = 15)	*p*-value
Age (years)			
Median	50	52	0.612
Range	40–70	37–80	
Histopathological type			
Squamous cell carcinoma	13	14	0.543
Adenocarcinoma	2	1	
Margin status			
Positive	2	2	0.828
Negative	2	1	
Unknown	11	12	
Parametrial status			
Positive	1	0	0.595
Negative	2	2	
Unknown	12	13	
Lymphovascular invasion			
Positive	2	3	0.536
Negative	3	1	
Unknown	10	11	
Lymph nodal status			
Positive	4	5	0.068
Negative	11	9	
Induction chemotherapy			
Given	4	5	0.232
Not given	11	10	
EBRT technique			
2D conventional	3	9	0.323
3D CRT	12	6	
EBRT dose			
45 Gy	12	7	0.124
46 Gy	2	3	
50 Gy	1	5	
Concurrent chemotherapy			
Yes	13	14	0.543
No	2	1	
RT duration (days)			
Median	56	58	0.247
Range	43–159	45–196	

ISBT, Interstitial Brachytherapy; IVBT, Intravaginal Brachytherapy; 3DCRT, 3-Dimensional Conformal Radiotherapy; EBRT, External Beam Radiotherapy.

**Fig. 1:**
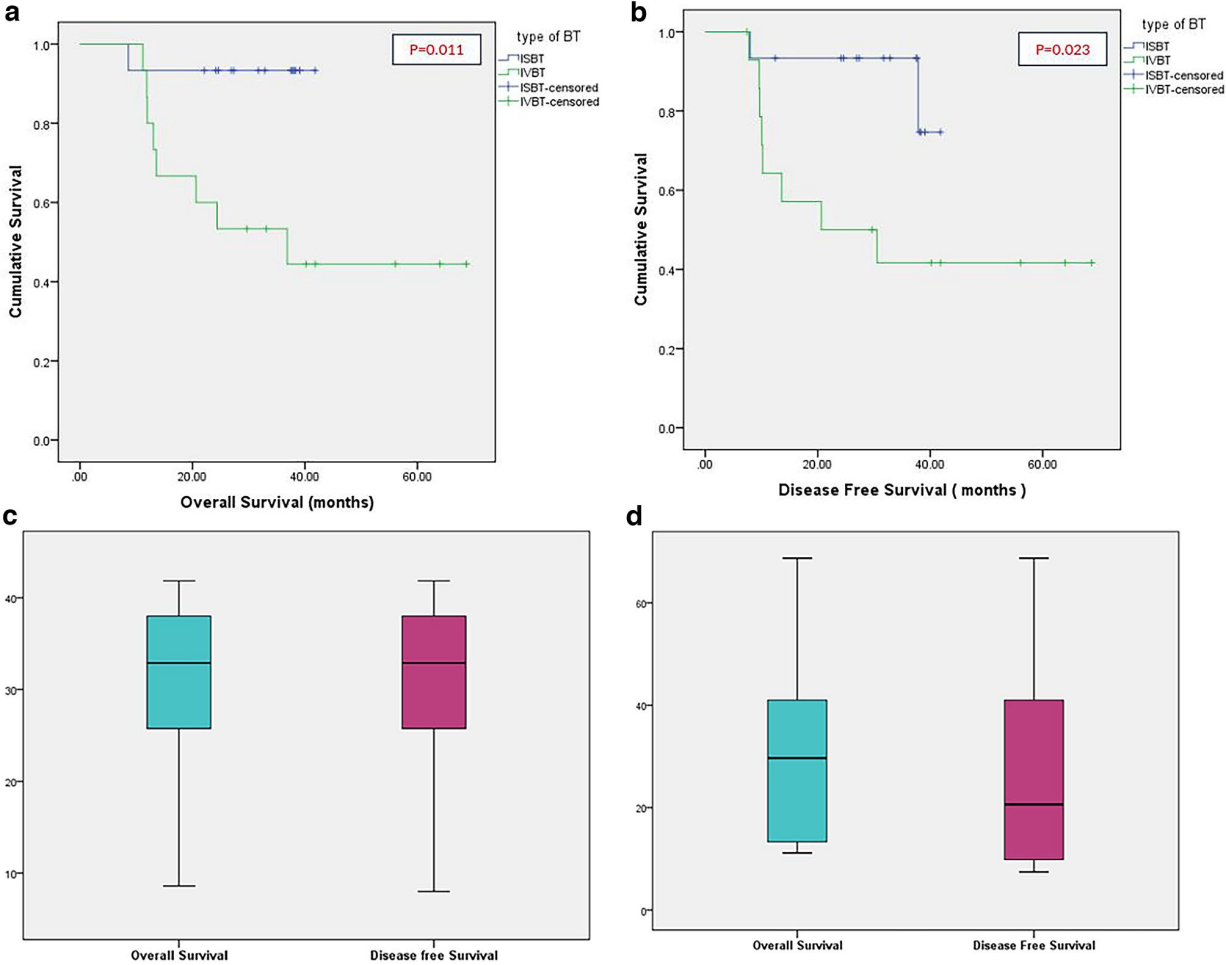
Kaplan-Meier graph comparing survival outcomes in IVBT and ISBT patients. (**a**) Overall Survival (OS); (**b**) Disease-Free Survival (DFS); (**c**) Distribution of OS and DFS for ISBT arm; (**d**) Distribution of OS and DFS for IVBT arm.

The treatment outcomes of all the patients are shown in Table 2. The median follow-up (FU) period was 24.3 months and 32.8 months in the IVBT and ISBT arms respectively. Fourteen patients in both the arms had complete clinical response at the time of BT. One patient in the ISBT arm had a stable disease while one patient in IVBT had a partial response. The local control rate was 60% in the IVBT arm and 93% in the ISBT arm (*p* = 0.031). Eight patients died due to disease in the IVBT arm whereas there was only one death in the ISBT arm (Table 3). Median OS and DFS in the IVBT arm were 36.9 and 20.6 months, respectively. The same could not be reached in the ISBT arm at the time of analysis. The 3-year OS for IVBT and ISBT arm was 54% and 93%, respectively (*p* = 0.011) (Fig. 1a). The 3-year DFS for IVBT and ISBT arm was 42% and 93%, respectively (*p* = 0.023) (Fig. 1b). The distribution of survival means for ISBT and IVBT is given in Fig. 1c and d.

**Table 2:** Treatment outcome of ISBT and IVBT.

Parameters	ISBT (*n* = 15)	IVBT (*n* = 15)	*p*-value
Follow-up duration (months)			
Median	32.88	24.37	0.558
Range	8.6–41.82	7.43–68.73	
Last follow up status			
Alive without disease	12	6	**0.038**
Alive with disease	1	0	
Lost on follow-up without disease	1	1	
Dead due to disease	1	8	
Overall survival (months)			
Median	NR	36.8	**0.011**
Mean	39.6	40.9	
Disease-free survival (months)			
Median	NR	20.6	**0.023**
Mean	38.8	37	
Recurrence pattern			
Local	1	3	**0.031**
Distant	0	3	
None	14	9	

ISBT, Interstitial Brachytherapy; IVBT, Intravaginal Brachytherapy.

**Table 3:** Acute and late toxicities from ISBT and IVBT.

Parameters	ISBT (*n* = 15)	IVBT (*n* = 15)	*p*-value
Acute Gastrointestinal toxicity			
Grade 0	2	8	0.067
Grade 1	11	6	
Grade 2	2	1	
Acute Bladder toxicity			
Grade 0	12	7	**0.052**
Grade 1	2	8	
Grade 2	0	0	
Grade 3	1	0	
Late Gastrointestinal toxicity			
Grade 0	9	9	0.721
Grade 1	5	4	
Grade 2	0	0	
Grade 3	0	0	
Grade 4	1	2	
Late Bladder toxicity			
Grade 0	9	9	0.484
Grade 1	4	6	
Grade 2	1	0	
Grade 3	1	0	
Late Rectal toxicity			
Grade 0	13	14	0.541
Grade 1	0	0	
Grade 2	1	0	
Grade 3	1	0	
Grade 4	0	1	

ISBT, Interstitial Brachytherapy; IVBT, Intravaginal Brachytherapy.

The median gap between surgery and registration in both the arms with us was 11.9 months. About 1/3rd of our patients had presented to us within 6 months of surgery. Patients who presented within 6 months of surgery had a mean OS of 38.4 months while those with late presentation had 35.6 months of mean OS (*p* = 0.863).

**Fig. 2:**
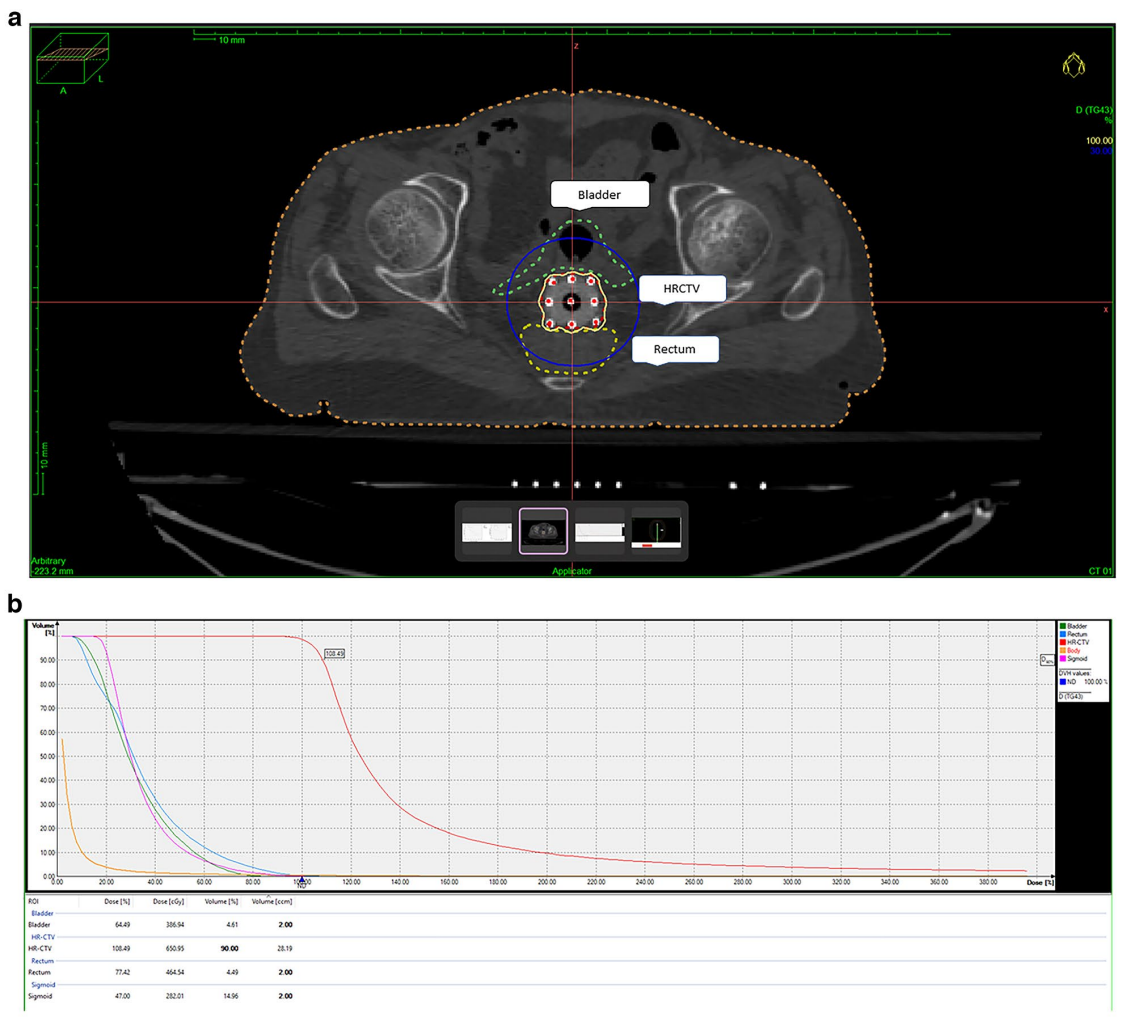
(**a**) Representative axial CT image showing the isodose distribution of 100% (yellow) dose in ISBT patient, prescribed to HRCTV and optimized by IPSA; (**b**) Dose-Volume Histogram for ISBT.

Acute and late toxicities in both arms were comparable (Table 3). Late bladder toxicity of grade 3 was seen in one patient of the ISBT arm in Fig. 2a. This patient had received 121% of the prescribed BT dose to D_0.1cc_bladder. One patient in the ISBT arm who had received 120% of the prescribed dose to 0.1 cc volume of rectum developed late rectal toxicity of grade 3 (Supplementary Appendix, Table 1). The dose-volume histogram (DVH) in Fig. 2b demonstrates the dose received by 2 cc of the volume of OARs with ISBT is within the normal limits as defined by ICRU 89.

## Discussion

We have compared the treatment outcomes of the two BT techniques in this study. To the best of our knowledge, this study is the first of its kind as IVBT has never been compared with ISBT in cervical cancer patients with residual/recurrent disease after inadvertent hysterectomy.

Earlier we used to treat these patients with concurrent chemo-radiation (CCRT) followed by IVBT but the survival outcome was disappointing. The cumulative EQD2 dose (EBRT with BT) with our dose schedule was 60.25 Gy which seems to be inadequate for patients treated with gross disease. However, a definitive BT dose schedule remains elusive for these patients, with doses in the literature ranging from 15 Gy to 50 Gy with LDR or HDR-BT (Supplementary Appendix, Table 2)^9–11,13,18–25^.

The dip in the isodose curve, due to self-absorption at the vaginal apex, is a known phenomenon in IVBT with a single-channel vaginal cylinder. The thickness of the vagina at the vaginal vault is about 2.5–2.9 mm^26,27 ^. Figure shows the fall in isodose coverage at the vault with an increase in the thickness of the vaginal wall. Since most of these patients presented to us after a long time after hysterectomy, the vaginal fibrosis might have developed resulting in increased thickness of the vaginal wall. Tissues treated with RT are known to develop edema. The tumor response to EBRT may not be uniform which may leave areas of disease not well appreciated on clinical examination. The above three reasons may together increase the thickness of the vaginal apex to about 1 cm. Therefore, we inserted the implant needles 2 cm beyond the vaginal apex or proximal extent of the residual disease (1 cm for offset and 1 cm for the thickness of the vaginal apex) and prescribed a dose of 4 fractions of 6 Gy with HDR to HRCTV with ISBT.

EQD2 in the ISBT arm was 76.25 Gy compared to 60.25 Gy in the IVBT arm. The increase in dose with ISBT seems to be one of the major reasons for significant improvement in local control of the disease. The dose optimization with ISBT allows better dose distribution to the target with the sparing of OARs with the potential of improved therapeutic ratio. Further increase in dose with IVBT would have increased the toxicity to the rectum and bladder. There is dose conformity and scope of dose optimization with ISBT. Therefore, we had decided to use ISBT to augment the dose so as to improve the local control and survival outcome.

In the majority of the earlier studies, RT was not combined with concurrent chemotherapy (Supplementary Appendix, Table 2)^9–11,13,18–25^. This could be one of the reasons for inferior local control and survival in these studies as compared to our study. RT with concurrent cisplatin has a proven role in intact cervical cancer^28^ as well as in patients with positive margins or positive lymph nodes after radical hysterectomy^29–31^. Based on the above evidence, we practice concurrent cisplatin with RT in all such cases.

Choudhary et al. retrospectively assessed the role of taxane-based induction chemotherapy followed by RT and BT in patients with inadvertent hysterectomy for invasive cervical cancer and reported that the patients treated with taxane-based induction chemotherapy had better survival outcomes than their counterparts^32^. Similarly, the study by Kim et al. evaluated consolidated chemotherapy using paclitaxel and carboplatin after concurrent CRT and found to be infeasible due to increased toxicity without clear survival benefits^33^. These findings align with the results of OUTBACK trial, further questioning the efficacy of additional chemotherapy in this context^34^. As proven by metaanalysis by Horweg et al. there was lack of significant survival benefit with addition of adjuvant chemotherapy after concurrent chemo-radiation in patients with cervical cancer^35^. In our study, survival outcome was better with upfront RT, regardless of the BT technique used, as compared to treatment with induction chemotherapy.

You et al. evaluated the effect of the interval between surgery and adjuvant therapy on the oncologic outcome for patients with early-stage cervical cancer. The authors reported that the 5-year OS and DFS were higher for the shorter interval than for the interval longer than 5 weeks^36^. In the study by Andras et al., the survival was worst for patients in whom the gap between surgery and RT was above six months^10^. Similarly, Davy et al. found that the patients who had R1 resection and were referred within 6 months for RT had better OS than those who were referred after 6 months of surgery (37.5% vs. 20%)^9^. We did not observe any significant difference in survival concerning the timing of the presentation of the patients to us.

Mahantsthetty et al. reported acute grade 3–4 gastrointestinal (GI), and genitourinary (GU) toxicities in 11%, and 9% of patients after inadvertent surgery for cervical cancer^37^. In our study, there was a trend towards higher acute grade 3 GU toxicity in the ISBT arm whereas none of the patients developed acute grade 3–4 GI toxicity. Despite receiving a higher dose, patients of the ISBT arm experienced comparatively less toxicity than their counterparts; nonetheless, this difference was not statistically significant.

## Conclusion

When compared to intravaginal brachytherapy boost, there was a significant improvement in the survival outcome with increase in total dose using interstital brachytherapy for patients presenting with residual or recurrent disease after inadvertent hysterectomy. However, to fully discern the extent of ISBT’s advantages, a meticulously designed prospective randomized study becomes imperative.

## Electronic supplementary material

Below is the link to the electronic supplementary material.

Supplementary Material 1

## Data Availability

Research data are available and will be shared upon request to the corresponding author.
