# Supplementary material for: Comparison of intravaginal and interstitial brachytherapy in cervical cancer after inadvertent hysterectomy: a retrospective study
Source: Sci Rep. 2025 Sep 30;15:33784. doi: 10.1038/s41598-025-99935-4 (PMC12484820; doi:10.1038/s41598-025-99935-4)
Supplement: Supplementary file 1 — Supplementary Material 1 [file 41598_2025_99935_MOESM1_ESM.docx]

| **Parameters** | **ISBT (n:15)** |
| --- | --- |
| **Mean Bladder dose (%)**  D2cc  D1cc  D0.1cc | 72.6  79.07  94.99 |
| **Mean rectal dose (%)**  D2cc  D1cc  D0.1cc | 81.65  91.17  121.66 |
| **Mean sigmoid dose (%)**  D2cc  D1cc  D0.1cc | 29.03  34.18  46.12 |
| **Mean HRCTV dose(%)**  D90  D100  V150 | 111.25  81.65  36.14 |

**Table 1: Dosimetric characteristics of ISBT arm**

**Table 2: Review of Literature**

| **Author** | **Year** | **No of patients** | **Patients with evidence of gross disease** | **Salvage treatment** | **EBRT dose** | **BT type** | **BT dose** | **Outcome**  **5-year OS** |
| --- | --- | --- | --- | --- | --- | --- | --- | --- |
| Davy et al [9] | 1977 | 74 | 74 | Group 1- stage I, free operative borders- EBRT+BT  Group 2- not free borders, referred within 6 months- EBRT+BT  Group 3- not free borders, referred after 6 months- EBRT+BT | 3000-5000 rads | LDR | - | 77%  37.5%  20% |
| Roman et al[7] | 1993 | 122 | 30 | EBRT + BT in margin negative or positive with no gross disease  EBRT alone in gross disease | No gross disease 45-55Gy  With gross disease :65Gy | LDR | 30-50Gy  none | Overall:65%  No gross disease: 75%  With gross disease:39% |
| Andras et al[8] | 1973 | 118 | 118 | Group 1- microscopic disease- BT alone  Group 2- gross disease in specimen- EBRT+BT  Group 3- cut through margin- EBRT +BT  Group 4- gross residual tumor- EBRT alone/ EBRT+BT  Group 5- gap between surgery and RT >6 months- EBRT alone/ EBRT+BT | -  2000-6000 rads  5000-6000 rads  5000-7000 rads  6000-7000 rads | LDR | 6000Rads  3000-3500 rads  3000-3500 rads  3000-3500 rads  2500-3000 rads | 96%  84%  87%  47%  37% |
| Cosbie et al [16] | 1963 | 175 | 99 | RT | - | - | - | Simple hysterectomy: 46%  Supravaginal hysterectomy:27% |
| Fang et al [17]  (Abstract only) | 1993 | 73 | 34 | Group A- without gross disease(n:32)  Group B – with gross disease (n:23)  Group C- recurrent tumor(n:11)  EBRT alone in 30 patients  EBRT + BT in 36 patients | EBRT alone:  45-50Gy + boost 30Gy  EBRT+BT: 45-50Gy | HDR | 30Gy | Overall: 67%  Group A: 81%  Group B: 56%  Group C: 45% |
| Choi et al [18] | 1997 | 64 | 13 | EBRT + BT  BT alone in 6 patients | 45-50Gy | HDR | 30-40Gy | Overall:75.8%  No gross disease:88.9%  With gross disease:27.9% |
| Crane et al [19] | 1999 | 18 | 4 | EBRT + BT in 12 patients  EBRT alone in 6 patients | 50- 54Gy  34-60Gy | LDR | Median dose: 50Gy @50cGy/hr | Overall: 93% |
| Huerta Bahena et al [20]  (Abstract only) | 2003 | 59 | 27 | RT | - | - | - | Overall: 59% |
| Chen et al [21] | 2003 | 54 | 29 | Group A- microinvasive disease- EBRT +BT  Group B- deep stromal invasion, LVI, positive or close margin, endometrial/ myometrial invasion, vaginal involvement- EBRT + BT | 55Gy | HDR | 15Gy | Overall: 88%  Group A: 95%  Group B: 82% |
| Hsu et al [22] | 2003 | 90 | 6 | Stage Ib- negative margin  Stage IIa- positive margin, no gross disease  Stage IIb- with gross disease | 45-50Gy  45- 50Gy  50-60Gy | LDR  HDR | 20-28Gy  Median: 21Gy | Overall: 85.5%  Stage Ib: 90%  Stage IIa: 76%  Stage IIb:50% |
| Garg et al [23] | 2004 | 40 | Gross disease (n): 10  Recurrent disease (n): 14 | RT | - | - | - | DFS  No gross disease:50%  With gross disease:26.3% |
| Saibish kumar et al [24] | 2005 | 105 | 81 | EBRT + BT | 46Gy | HDR | 19Gy | Overall: 55.2%  No gross disease:79.2%  With gross disease:48.2% |
| **Present study** | 2024 | 30 | 30 | EBRT+ concurrent CT +BT | 45-50Gy | HDR | IVBT:12Gy  ISBT:24Gy | 3-year OS:54%; DFS:42%  3-year OS:93%; DFS:93% |
